# A New Pathogenic Missense Variant in a Consanguineous North-African Family Responsible for a Highly Variable Aceruloplasminemia Phenotype: A Case-Report

**DOI:** 10.3389/fnins.2022.906360

**Published:** 2022-05-02

**Authors:** Hervé Lobbes, Quitterie Reynaud, Sabine Mainbourg, Claire Savy-Stortz, Martine Ropert, Edouard Bardou-Jacquet, Stéphane Durupt

**Affiliations:** ^1^Service de Médecine Interne, Hôpital Estaing, CHU de Clermont-Ferrand, Clermont-Ferrand, France; ^2^SIGMA Clermont, Institut Pascal, CHU Clermont-Ferrand, Université Clermont Auvergne, CNRS, Clermont-Ferrand, France; ^3^Département de Médecine Interne et Centre de Référence Mucoviscidose, Centre Hospitalier Lyon Sud, Hospices Civils de Lyon, Pierre-Bénite, France; ^4^Research on Healthcare Performance (REHSAPE), INSERM U1290, Université Claude Bernard Lyon 1, Lyon, France; ^5^Equipe Evaluation et Modélisation des Effets Thérapeutiques, UMR 5558, Laboratoire de Biométrie et Biologie évolutive, CNRS, Université Claude Bernard Lyon 1, Villeurbanne, France; ^6^Médecine interne et médecine vasculaire, Groupe Hospitalier les Portes du Sud, Vénissieux, France; ^7^INSERM, University of Rennes, INRAE, UMR 1241, AEM2 Platform, Nutrition Metabolisms and Cancer (NuMeCan) Institute, Rennes, France; ^8^Department of Biochemistry, CHU de Rennes, Rennes, France; ^9^Liver Disease Department, French Reference Center for Hemochromatosis and Iron Metabolism Disease, CHU de Rennes, Rennes, France; ^10^INSERM, CIC141, CHU de Rennes, Rennes, France

**Keywords:** aceruloplasminemia, ferroxidase, iron overload, neurodegenerative disease, genetic variation

## Abstract

Aceruloplasminemia is a rare autosomal recessive inherited disorder. Mutations in the ceruloplasmin gene cause depressed ferroxidase activity leading to iron accumulation. The clinical phenotype is highly variable: anemia, retinopathy, diabetes mellitus, psychiatric disorders, and neurological symptoms including parkinsonian disorders and dementia are the main features of this disease. Characterized by high serum ferritin with low transferrin saturation, aceruloplasminemia uniquely combines brain, liver and systemic iron overload. We report here four new cases of aceruloplasminemia in a consanguineous North-African family. Genetic sequencing revealed a homozygous missense variant c.656T>A in exon 4 of the ceruloplasmin gene, which had been described previously as of “unknown significance” in the dbSNP database and never associated with ACP in the HGMD database. Ferroxidase activity was strongly depressed. Clinical manifestations varied among cases. The proband exhibited mild microcytic anemia, diabetes mellitus, psychosis and parkinsonism, whereas the other cases were asymptomatic or mildly anemic, although high serum ferritin and brain iron deposition were documented in all of them. Therapeutic management was complex. The proband started deferoxamine treatment when already symptomatic and he rapidly declined. In the asymptomatic cases, the treatment was associated with poor tolerance and was discontinued due to anemia requiring red blood cell transfusion. Our series illustrates the need for new therapeutic approaches to aceruloplasminemia.

## Introduction

Hereditary aceruloplasminemia (ACP, OMIM #604290) is a rare autosomal recessive neurodegenerative disorder caused by an absent or depressed ferroxidase function of ceruloplasmin (CP) due to mutations in the CP gene (Miyajima, 2015; Pantopoulos, 2018). Two isoforms of CP are synthetized according to an alternative splicing: the soluble CP is synthetized by hepatocytes and bound 90% of copper in the plasma (Hellman and Gitlin, 2002). The other isoform is a glycosylphosphatidylinositol (GPI) anchored membrane form, involved in a crucial iron flux regulation through its ferroxidase function. In ACP, the impaired ferroxidase function of CP decreases efflux of iron stores both through diminished conversion of ferrous iron (Fe^2+^) to ferric iron (Fe^3+^) (Lobbes et al., 2020). Ferroxidase activity is mandatory because only Fe^3+^ can be incorporated into plasma transferrin and delivered to other cells: as such, iron bioavailability is decreased in ACP. Neurons requiring iron for the synthesis of neurotransmitters increase the uptake of alternative iron (non-transferrin-bound-iron) which is highly toxic and increases neuronal death (Breuer et al., 2000). Moreover, the increased iron stores in brain tissues are in turn responsible for an intense oxidant stress: free iron generates free radicals (Fenton reaction) leading to neuronal damage (Patel et al., 2002). Macrophages, hepatocytes, pancreatic and retinal epithelial cells express GPI-anchored membrane CP isoform, leading to the main clinical and biological manifestations of ACP (Kono et al., 2010): mutant CP fails to stabilize ferroportin on the cells surface leading to intracellular iron accumulation.

Epidemiologic data derive from estimations in the Japanese population where the estimated prevalence in non-consanguineous unions is 1 per 2 000 000 (Miyajima et al., 1999). About 130 cases have been reported worldwide (Vroegindeweij et al., 2017; Marchi et al., 2019). The main clinical manifestations of ACP are retinal degeneration, diabetes mellitus (Vroegindeweij et al., 2015), and neurologic disorders including cerebellar signs and involuntary movements with an onset between 40 and 60 years of age (McNeill et al., 2008). Diabetes mellitus has been showed to precede neurological disorders in the majority of the patients, with a median age of 38.5 years (Vroegindeweij et al., 2015): the underlying mechanism is poorly understood but might be due to the iron toxicity (Pelucchi et al., 2018). However, the reduction of islet pancreas β cells contrasts with the preferred accumulation of iron in the exocrine portion of pancreas (Morita et al., 1995). Psychiatric disorders affect half of Caucasian ACP patients and can be the first neurological manifestation of ACP (Vroegindeweij et al., 2019).

High serum ferritin (SF) levels (up to 3,000 μg/L), contrasting with low or normal transferrin saturation (TSAT) levels and mild microcytic anemia, are the earliest features of ACP. Indeed, although TSAT is not a quantitative index for iron overload, its elevation is a marker of increased duodenal iron absorption and increased iron release from macrophages. Thus, high SF with normal TSAT is suggestive of diseases due to inefficient iron export such as ferroportin disease or ACP over hereditary hemochromatosis (Piperno, 2013). Biochemical signs of the disease may be present several years before the onset of clinical symptoms, which typically appear during the fourth of fifth decade. The diagnosis of ACP is based on the association of evocative clinical and biochemical features and confirmed by a decreased serum CP levels and low ferroxidase activity (Marchi et al., 2019).

ACP treatment is mainly based on iron chelation, using either deferoxamine, deferasirox or deferiprone. The therapy of choice remain to be determined and no recommendations are available to date. Iron chelation is efficient to decrease the systemic iron overload but less or not effective on neurological symptoms, glucose metabolism and retinopathy. Zinc (Kuhn et al., 2007) and minocycline (Hayashida et al., 2016) has been used in ACP, based on iron chelation, antioxidant properties and inhibition iron absorption but evidence is limited to case report. Finally, fresh frozen plasma infusion (Piperno and Alessio, 2018) to increase CP level and to restore the ferroxidase function has been proposed, through fresh frozen plasma infusions but the risk of repeated transfusion limits this approach.

Recent publications highlight a very high heterogeneity in clinical and biological manifestations of ACP (Pelucchi et al., 2018; Vila Cuenca et al., 2020). Most cases are due to homozygous mutations in the CP gene, but compound heterozygosity has also been reported (Lindner et al., 2015; Yamamura et al., 2018; Stelten et al., 2019; Aydemir et al., 2020; Ondrejkovičová et al., 2020; Salsone et al., 2021; Xiao et al., 2021; Xu et al., 2021). More than 70 mutations, mostly missense and frameshift, less frequently splicing and non-sense have been reported to date. We report here the case of four members of a consanguineous North-African family carrying the same missense CP variant, previously described as of “uncertain significance” and not recorded in the HGMD 2019 database (HGMD^®^ gene result).

## Patients and Methods

The patients were diagnosed and followed in our referral center for rare iron overload disease (Hospices Civils de Lyon, Lyon Sud University Hospital). All the patients or their legal representative gave written informed consent for scientific publication and genetic testing in accordance with the Declaration of Helsinki. In compliance with French regulations, ethical approval for scientific publication was waived.

### Biochemical and Genetic Testing

Routine tests were performed in our center: ferritin (chemiluminescent microparticle immunoassay, Cobas8000 Roche©, normal range 13–76 μg/L), iron (FerroZine method, Roche©, normal range 12.5–25 μmol/L), transferrin (immunoturbidimetry, Cobas8000 Roche©, normal range 2–3.6 g/L) and transferrin saturation, and complete blood count.

Specific analyses including for serum ceruloplasmin, ferroxidase activity and genomics were performed centrally at the French national reference center for rare iron overload disease of genetic origin (Centre Hospitalier de Pontchaillou, Rennes, France). Ceruloplasmin assay was performed by immunoturbidimetry (Roche©, normal range 0.16–0.45 g/L). Ferroxidase activity of ceruloplasmin was determined using the colorimetric method by Erel (1998) (normal range for ferroxidase I: 440–560 IU/L in men, 550–750 IU/L in premenopausal women and 490–650 IU/L in menopausal women).

Genetic variants were reported following the Human Genome Variation Sequence (HGVS) nomenclature: sequence reference NM_000096.3 for the CP transcript variant. The analysis was performed using the Sanger method.

### Brain and Liver Imaging

Brain iron accumulation (T2, T2^*^ and T2 fast spin echo) and liver iron concentration using the method of Gandon et al. (2004) were evaluated for each patient by magnetic resonance imaging at 1.5 T and centrally assessed in our center (McNeill et al., 2008).

## Results

**Figure 1:**
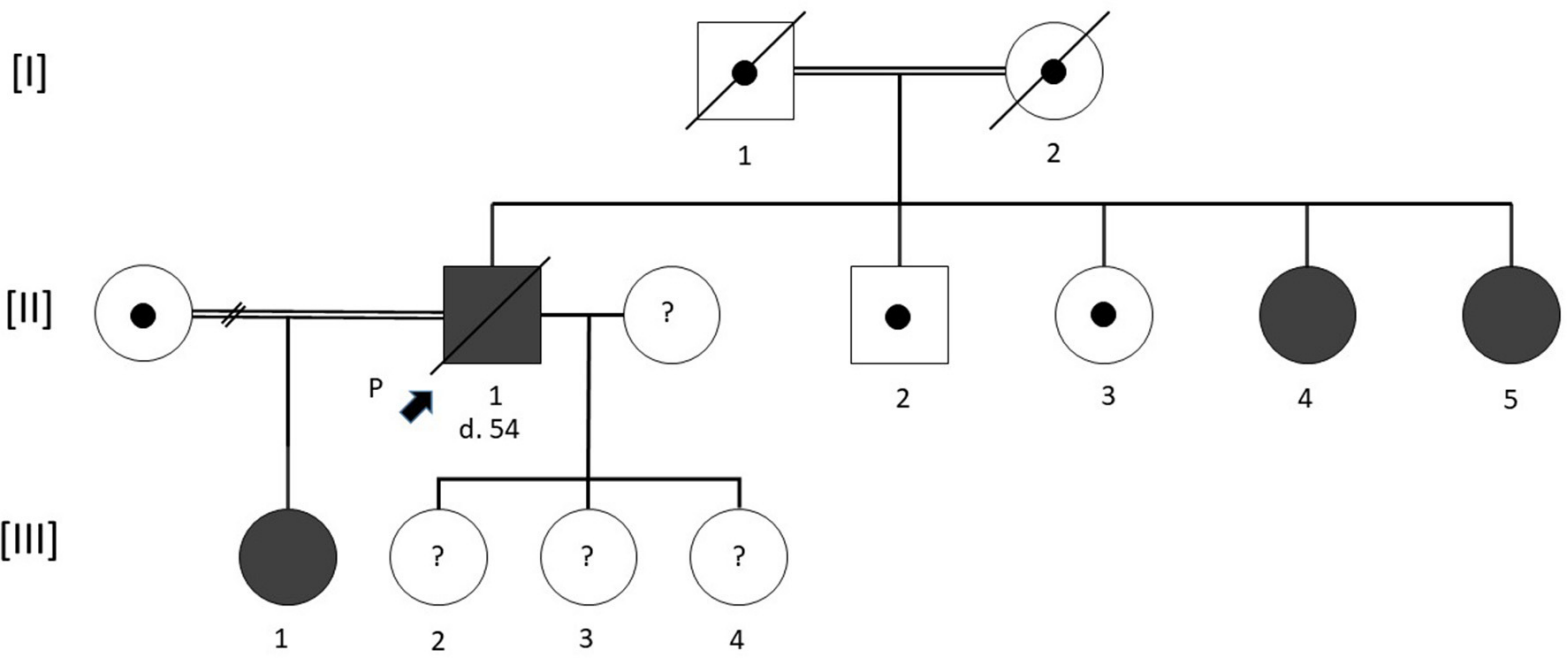
Pedigree of the family. Squares indicate male and circles female. The proband is indicated by a solid black arrow. Filled symbols represent patients carrying the same homozygous p.Val219Glu mutation in the ceruloplasmin gene. A black circle inside a symbol indicates an obligatory carrier who was not investigated by our team. Deceased subjects are shown by a transversal bar crossing the symbol. Age of death is indicated by a number preceded by the letter d when known. A double horizontal bar linking two symbols represents a consanguineous union. A horizontal link crossed by a double transversal crossbar between two individuals indicates a dissolved union. A question mark inside a symbol indicates unknown phenotype and genotype.

Figure 1 shows the pedigree of the family according to the standardized human pedigree nomenclature (Bennett et al., 2008).

### The Proband Case: Clinical, Biological, and Imaging Characteristics

#### Medical History

The proband (II.1) was an obese 50-year-old man born in Algeria. He had a history of type 2 diabetes mellitus for 5 years complicated by diabetic nephropathy with chronic kidney disease stage 3a. He was treated for mild hypertension complicated by hypertrophic cardiomyopathy. At age 46 years, he was treated for severe depression. Four years later, his family reported cognitive decline associated with behavioral changes comprising alternating phases of apathy, aggressiveness, and psychosis, and he was referred to our local department.

Physical examination did found no extrapyramidal symptoms or ataxia. Ophthalmological examination found no evidence of retinopathy. A complete blood count found a mild microcytosis (mean corpuscular volume 78 fL) without anemia. Subsequent evaluation revealed elevated serum ferritin (SF 955 μg/L) with low iron serum (8 μmol/L) and mildly decreased transferrin saturation (TSAT 16%) without inflammatory syndrome (C reactive protein 4.6 mg/L) and no sign of thalassemia.

The concomitant occurrence of diabetes mellitus, mild microcytosis with increased SF and low TSAT, and behavioral changes with incipient psychopathology suggested ACP. Total blood copper was markedly decreased (1.8 μmol/L) and serum ceruloplasmin was undetectable (<0.2 μmol/L). Serum ferroxidase activity was strongly depressed (total ferroxidase activity 31 IU/L, normal range 700–800 IU/L, ferroxidase I activity 7 IU/L, normal range 550–750 IU/L).

#### Brain and Liver Imaging

**Figure 2:**
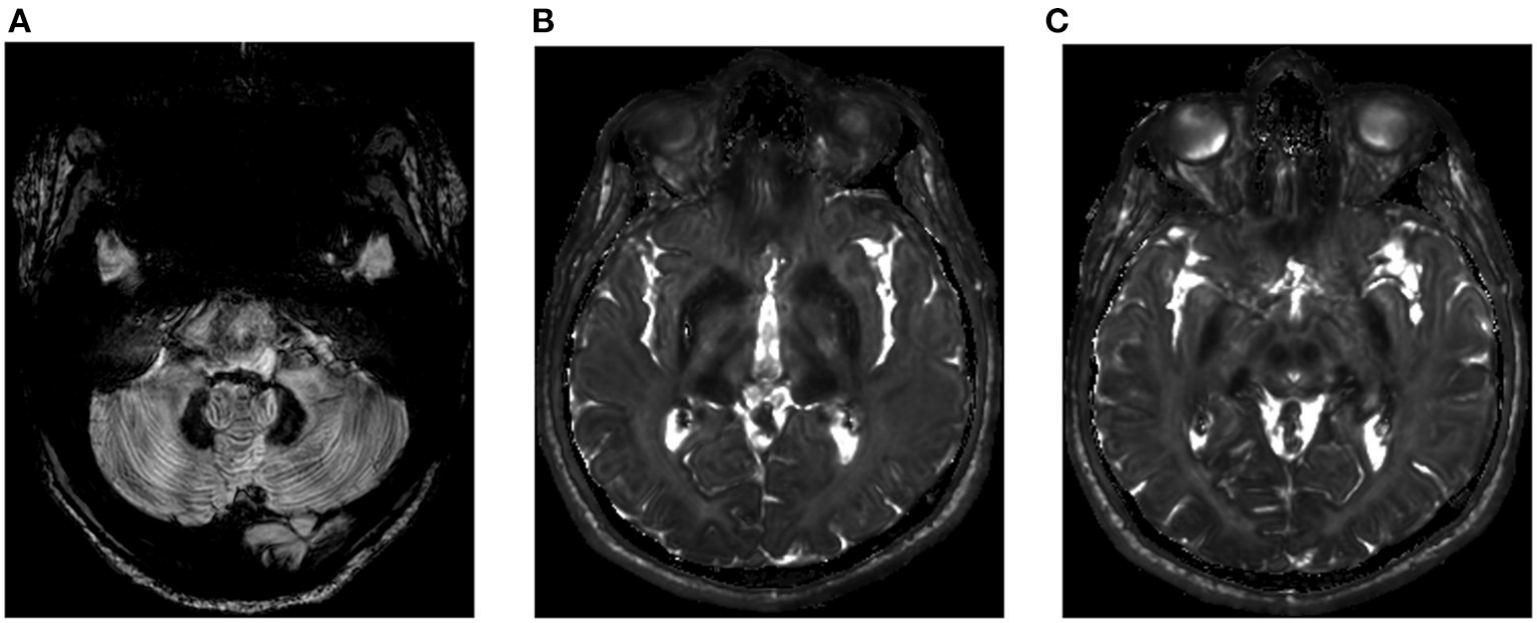
Brain imaging (Spin echo T2 weighted) of the proband. **(A)** Hypointensity of the caudate and thalamus nuclei, **(B)** hypointensity of the red nuclei, and **(C)** hypointensity of the dentate nuclei and cerebellar atrophy.

Hepatic iron content was 300 μmol/g dry weight (normal <36 μmol/g dry weight). Iron deposition (Figure 2) in the brain was found in the red nuclei, caudate nuclei, the thalamus and the cerebellar dentate nuclei. The cerebellum also exhibited a marked atrophy but the patient showed no clinical evidence of cerebellar syndrome.

#### Genetic Testing

Using Sanger sequencing, we identified a homozygous missense variant c.656T>A (p.Val219Glu) in exon 4 of the CP gene, confirming the diagnosis of ACP. Interestingly, this variant had been described previously as of “unknown significance” in the dbSNP database and never associated with ACP in the HGMD database. We did not perform any additional genetic testing for others iron overload diseases as the clinical and biological features were highly suggestive for ACP.

### Follow-Up of the Proband

The patient received subcutaneous deferoxamine 20 mg/kg 5 days a week from age 51. Because of severe impairment of renal function 6 months after initiation of the treatment, the dosage was reduced to 20 mg/kg three times a week.

Despite treatment efficacy on iron variables, cognitive impairment gradually increased. One year after treatment initiation, he developed severe cerebellar ataxia with multiple falls. Extrapyramidal syndrome, asymmetric rigidity, and dyskinesia of the left arm and limb rapidly worsened. Three years after the diagnosis, the patient had severe dementia. He died at age 54 years from infectious post-operative complications of bladder surgery for neurogenic bladder dysfunction.

### Family Testing: High Heterogeneity Phenotypes

Table 1 shows the clinical and biological characteristics of the cases evaluated in our referral center. The proband's parents (consanguineous union) were deceased with no diabetes mellitus history or known neurocognitive impairment. Unfortunately, no biological data were available to search for anemia or microcytosis. The proband had one brother (II.2) and one sister (II.3) living abroad with whom he had no contact and were not investigated. Two sisters (II.4 and II.5) and a daughter (III.1) from a first union (consanguineous union) living in France were seen in our center. The proband's three other female children were underage at the time of diagnosis and could not be examined (refusal of their parents), but were apparently asymptomatic.

**Table 1:** Clinical, biological, and radiological findings in the proband's family.

**Characteristics**	**Proband (II.1)**	**II.4**	**II.5**	**III.1**
**Clinical**				
Age	50	52	53	20
Sex	Male	Female	Female	Female
Diabetes mellitus	Yes	No	No	No
Neurological involvement	Extrapyramidal syndrome, cerebellar ataxia	No	No	No
Psychiatric disorders	Mixed anxiety depressive disorder	No	No	Anxiety
Retinopathy	No	No	No	No
**Biological**				
Hemoglobin (g/dL) (Range: 13–16.5)	13.3	12.5	11.0	10.9
MCV (fL) (Range: 80–100)	78	83	80	76
Ferritin (μg/dL) (Range: 13–76)	955	1,188	1,050	475
Serum iron (μmol/L) (Range: 12.5–25)	8	7	6.4	3.3
TSAT (%) (Range: 16–40)	16	35	14.5	5
CP (μmol/L) (Range: 0.16–0.45)	Undetectable	Undetectable	Undetectable	Undetectable
Ferroxidase I activity (IU/L) (Range: 550–750)	7	NA	NA	9
**Iron deposition**				
HIC μmol/g dry weight (Normal <36 μmol/g)	300	330	300	290
Brain iron MRI	Caudate nuclei, thalamus, red nuclei, dentate nuclei	Red nuclei, basal ganglia, thalamus	Red nuclei, basal ganglia	Red nuclei, basal ganglia, thalamus, cerebral cortex
Cerebellar atrophy	Yes	No	No	No

*All the patients carried the same homozygous mutation c.656T>A*.

*CP, ceruloplasmin; HIC, hepatic iron content; MCV, mean corpuscular volume; MRI, magnetic resonance imaging; NA, not available; TSAT, transferrin saturation*.

Both the proband's sisters (II.4 and II.5) received deferoxamine 20 mg/kg 5 days a week. Tolerance was acceptable apart from the occurrence of a mild microcytic anemia 3 months after treatment initiation. They returned to their country of birth, continuing the treatment with acceptable clinical tolerance. Five years after the diagnosis, they had developed no psychiatric or neurologic disorders.

The proband's daughter (III.1) also received deferoxamine, but anemia quickly worsened requiring red blood cell transfusion, and so treatment was ceased. Five years after the diagnosis and 3 years after discontinuation of deferoxamine, she still had mild microcytic anemia (hemoglobin 11.6 g/dL, MCV 76 fL). Ferritin dropped to 286 μg/L. At the last visit in February 2022, she was asymptomatic. Deferiprone will be the next line of treatment if she agrees to resume iron chelation.

## Discussion

We report four cases of ACP in non-Japanese patients with a new missense variant in the CP gene previously reported as of unknown significance. Several studies recently reported new pathogenic variants in this rare disease (Lindner et al., 2015; Ondrejkovičová et al., 2020; Vila Cuenca et al., 2020).

The cases in this consanguineous family are highly illustrative of the clinical and biological heterogeneity of the disease's phenotype. This report also highlights the therapeutic challenge of iron chelation. A recent analysis of aggregated cases suggested that treatment might delay the onset of neurological manifestations (Vroegindeweij et al., 2020). In our series, the proband started treatment before the onset of neurologic disorders but he rapidly experienced cerebellar involvement and developed dementia. We did not perform any brain MRI to study iron deposition evolution after iron chelation therapy, and to date this information is scantly reported in the literature. We hypothesize that the psychiatric symptoms might be a marker of poor prognosis in ACP as they may reflect the onset of neurological damage.

There are no established recommendations in ACP treatment. The therapeutic strategy is based on iron chelation with phlebotomy (Vroegindeweij et al., 2020) or iron chelators. In our case series, the choice of deferoxamine was based on several considerations: due to its hexadentate structure allowing to bind 1 iron atom at a 1:1 mol ratio, deferoxamine is reported as the most powerful iron chelator. Moreover, deferoxamine is able to bind the iron released by old red blood cell, allowing to prevent the oxidative effects or non-bound iron. Furthermore, the onset of cognitive impairment in our patient made us fear non-compliance with oral treatment. Its use has been associated with a decreased iron brain deposition and liver concentration (Loréal et al., 2002) but tolerance is poor as illustrated in our cases (kidney injury, increased anemia).

Deferiprone is on oral treatment reported as effective (Bove and Fasano, 2015) in ACP. This low-weight lipophilic agent represents an attractive alternative therapy, because it can cross the blood-brain barrier and could prevent brain iron deposition and neuronal damage. Deferiprone has been used in monotherapy or combined with deferoxamine (Badat et al., 2015) or fresh frozen plasma (Poli et al., 2017). However, reduced access to plasma and the risk of viral transmission limits its use given the suspensive nature of such treatment. Deferasirox has also been used in ACP (Suzuki et al., 2013; Tai et al., 2014) with success on hepatic iron mobilization but no effect on brain iron accumulation (Finkenstedt et al., 2010).

The proband's daughter (III.1) discontinued iron chelation because of increased anemia requiring transfusion, illustrating the difficulty of treatment in asymptomatic and presymptomatic patients. A recent study in mouse models showed that intraperitoneal CP replacement therapy was effective: the authors reported decreased motor incoordination associated with decreased neuronal death (Zanardi et al., 2018). This strategy might offer a major therapeutic hope for a debilitating and fatal disease (Piperno and Alessio, 2018).

The description of new mutations in non-Japanese patients is crucial for future pathophysiological studies on ACP. The dissemination of knowledge about this rare disease will help physicians diagnose the disease early and start iron chelation therapy before brain iron accumulation and neuronal damage occur.

## Data Availability Statement

The original contributions presented in the study are included in the article, further inquiries can be directed to the corresponding author/s.

## Ethics Statement

Ethical review and approval was not required for the study on human participants in accordance with the local legislation and institutional requirements. The patients/participants provided their written informed consent to participate in this study. Written informed consent was obtained from the individual(s) for the publication of any potentially identifiable images or data included in this article.

## Author Contributions

HL and SD conceptualized the study. HL, QR, CS-S, MR, EB-J, and SD were responsible for clinical and biological investigation. HL drafted the initial manuscript. All authors reviewed and revised the final manuscript.

## Conflict of Interest

The authors declare that the research was conducted in the absence of any commercial or financial relationships that could be construed as a potential conflict of interest.

## Publisher's Note

All claims expressed in this article are solely those of the authors and do not necessarily represent those of their affiliated organizations, or those of the publisher, the editors and the reviewers. Any product that may be evaluated in this article, or claim that may be made by its manufacturer, is not guaranteed or endorsed by the publisher.
