# Supplementary material for: A New Pathogenic Missense Variant in a Consanguineous North-African Family Responsible for a Highly Variable Aceruloplasminemia Phenotype: A Case-Report
Source: Front Neurosci. 2022 May 2;16:906360. doi: 10.3389/fnins.2022.906360 (PMC9108494; doi:10.3389/fnins.2022.906360)
Supplement: Supplementary file 1 [file Data_Sheet_1.PDF]

CARE
case report guidelines

CARE Checklist of information to include when writing a case report

(cc) BY-NC-ND

Topic	Item	Checklist item description	Reported on Line
Title	1	The diagnosis or intervention of primary focus followed by the words "case report"	in the title
Key Words	2	2 to 5 key words that identify diagnoses or interventions in this case report, including "case report"	24 and 25
Abstract (no references)	3a	Introduction: What is unique about this case and what does it add to the scientific literature?	34 and 35
	3b	Main symptoms and/or important clinical findings	36 and 37
	3c	The main diagnoses, therapeutic interventions, and outcomes	32 and 33
	3d	Conclusion—What is the main "take-away" lesson(s) from this case?	39 to 43
Introduction	4	One or two paragraphs summarizing why this case is unique (**may include references**)	63 to 65
Patient Information	5a	De-identified patient specific information.	95
	5b	Primary concerns and symptoms of the patient.	97 to 105
	5c	Medical, family, and psycho-social history including relevant genetic information	95 to 97
	5d	Relevant past interventions with outcomes	95 to 97
Clinical Findings	6	Describe significant physical examination (PE) and important clinical findings.	101 and 102
Timeline	7	Historical and current information from this episode of care organized as a timeline	123 to 130
Diagnostic Assessment	8a	Diagnostic testing (such as PE, laboratory testing, imaging, surveys).	73 to 89
	8b	Diagnostic challenges (such as access to testing, financial, or cultural)	not applicable
	8c	Diagnosis (including other diagnoses considered)	118 to 121 and figure 1
	8d	Prognosis (such as staging in oncology) where applicable	not applicable
Therapeutic Intervention	9a	Types of therapeutic intervention (such as pharmacologic, surgical, preventive, self-care)	123
	9b	Administration of therapeutic intervention (such as dosage, strength, duration)	123 to 126
	9c	Changes in therapeutic intervention (with rationale)	126 to 130
Follow-up and Outcomes	10a	Clinician and patient-assessed outcomes (if available)	130 and 132 to 148
	10b	Important follow-up diagnostic and other test results	111 to 116 and table 1
	10c	Intervention adherence and tolerability (How was this assessed?)	124 and 132 to 148
	10d	Adverse and unanticipated events	144 and 145
Discussion	11a	A scientific discussion of the strengths AND limitations associated with this case report	158
	11b	Discussion of the relevant medical literature **with references**.	156, 163, 177
	11c	The scientific rationale for any conclusions (including assessment of possible causes)	178 to 184
	11d	The primary "take-away" lessons of this case report (without references) in a one paragraph conclusion	182 to 184
Patient Perspective	12	The patient should share their perspective in one to two paragraphs on the treatment(s) they received	patient deceased
Informed Consent	13	Did the patient give informed consent? Please provide if requested	Yes ☒ No ☐
